# Impact of litter on femur and tibial morphology, bone biomechanics, and leg health parameters in broiler chickens

**DOI:** 10.5713/ab.22.0335

**Published:** 2023-02-27

**Authors:** Komal Khan, Mehmet Kaya, Evrim Dereli Fidan, Figen Sevil Kilimci

**Affiliations:** 1 Department of Basic Sciences, University of Veterinary and Animal Sciences (sub-campus Jhang), Lahore 35200, Pakistan; 2 Department of Animal Science, Faculty of Veterinary Medicine, Aydın Adnan Menderes University, Aydın 09016, Turkey; 3 Department of Veterinary Anatomy, Faculty of Veterinary Medicine, Aydın Adnan Menderes University, Aydın 09016, Turkey

**Keywords:** Biomechanics, Bone, Broiler, Femur, Growth, Litter, Tibia

## Abstract

**Objective:**

In this study effects of three types of beddings on broiler leg health and bone biomechanics were evaluated.

**Methods:**

A total of 504 male chicks (Ross 308) were randomly placed on three beddings (4 replicates/group; 42 birds/pen), zeolite-added litter (ZL), plastic-grid flooring (PF), and wood shavings (WS). On day 42, chickens were weighed, slaughtered, and samples (bone, muscle, and drumstick) were collected. Bones were subjected to leg health tests, morphometric measurements, biomechanical testing, and ash analysis.

**Results:**

Broilers in PF and WS groups showed higher live weight than the ZL group (p<0.001), and the incidence of tibial dyschondroplasia (TD) and varus valgus deformity due to distal bending was significantly higher in PF (p<0.001). Multinomial logistic regression showed that bedding has a significant (p = 0.038) contribution toward the development of TD. Tibial strength (p = 0.040), drumstick width (p = 0.001), and total femur and epiphyseal ash contents (p = 0.044, 0.016) were higher in the ZL group. Chicken live weight was correlated with tibial length and weight (r = 0.762, 0.725).

**Conclusion:**

Flooring and the type of bedding material directly affect broiler bone length, strength and leg health. Plastic bedding improves the slaughter weight of chickens on the expense of leg deformities, and zeolite litter improves leg health and bone strength.

## INTRODUCTION

Poultry meat is a cheap source of protein and is eaten worldwide; it has low cholesterol levels and is market ready within a few weeks of hatching [1]. Broiler farming involves intensive feeding and exponential growth, continuous excretion of droppings, and production of toxic gases. So, the bedding/litter material is a critical concern as it directly affects the productivity and well-being of chickens. The most common litter for broiler rearing is pine shavings, paper, rice husks, sawdust, and leaves [2]. Zeolites (group of hydrated aluminosilicates of alkali and alkaline earth cations) are used along with other litter material due to the excellent absorption properties of moisture and obnoxious gases [3] and the benefits of reducing ammonia emissions.

Poultry farmers manage shed floorings and litter for a healthy environment with precise humidity. Conversely, poor litter management negatively affects birds' welfare through shortcomings like toxicity, scarcity, particle size, dust, dampness, microbial or mold growth, caking, and leg/foot/ammonia problems [4]. Lameness is a seriously growing welfare problem in fast-growing broilers, and it causes restricted activity with a wide range of leg disorders, i.e., tibial dyschondroplasia (TD), varus-valgus (VV) deformity, foot pad dermatitis (FPD) [5]. Recently, the type of flooring in broiler sheds has been given priority so that broilers' feed conversion ratio (FCR) and overall skeletal health are not overlooked.

Husbandry practices can change physiological measures of leg muscles and bone dimensions in broilers. Recently, attempts are underway in the form of various perches and lightning schedules to avoid deficiencies in the bedding materials [6]. Similarly, plastic-grid floors with different sizes and shapes of perforated designs are also one of these innovations. Such flooring provides better air circulation and prevents birds' legs from directly contacting litter [7].

The poultry shed environment directly impacts bone health parameters like weight, length, diameter, cortical thickness, and strength [8,9]. Similarly, bone quality decreases with higher growth rates [10], and the type of flooring can directly affect the bone length and breaking strength [11]. However, the relationship between slaughter weight, bone quality, and litter type is not well established. Given the above reasons, the main objective of this study was to compare three types of litter materials (zeolite and plastic-grid flooring as litter along with wood shavings) to determine whether the live weight of modern broilers correlates to their bone properties/features and if these litter materials influence broilers' performance, leg health, and bone biomechanical properties.

## MATERIALS AND METHODS

### Animals and experimental design

All experiments followed the ADU (Adnan Menderes University) Institutional Guidelines for the care and use of experimental animals no. DR/377, Vide. No. 27-8-2014. In the study, a total of 504-day-old male commercial broiler chicks (Ross 308) were housed in three experimental groups consisting of 12 replicates; each group consisted of 4 replicates. The experimental design is elaborated in Table 1. The first group was reared on zeolite bedding material (6 kg/m^2^ zeolite + 5 cm layer of pine wood shaving), the second group was provided plastic-grid floor (height, 5 cm; size, 50×50 cm; openings, 2×2 cm), and the third group was designated standard pine wood shaving litter material (5 cm layer of wood shavings).

### Sample collection and measurements

On day 42nd of the study, 120 birds (40 birds per group; 10 birds/each replicate) were randomly selected and weighed to get live weight (g) before slaughtering by severing their jugular veins. The live weight was classified as light (1,500 to 2,000 g), medium (2,001 to 2,500 g), and heavy (2,501 to 3,000 g).

A total of 240 femur and tibia bones of the right and left limbs each were removed. Afterwards, the soft tissues (muscles, ligaments, nerves, and vessels) were cleaned off by gentle dissection, and the bones were wrapped in sterile saline-soaked gauze, sealed in air-tight plastic bags and stored at −20°C until further processing. Bones were thawed up to 20°C for at least 10 to 20 minutes in normal saline, and measurements were made as described below (right tibias were used for TD scoring, VV deformity and biomechanics; morphometric measurements were taken on all the right bones; left femurs and tibias were used for ash and mineral analysis).

### Tibial dyschondroplasia scoring

At the end of the three-point testing, all right tibias were subjected immediately to mid-line slicing using an electric saw. The proximal growth plate of the tibia was cut open to assigning a score for TD (0 = no visual signs of TD; 1 = small cartilage lesion; 2 = large cartilaginous plug in the growth plate) [5].

### Varus-valgus deformation (VV deformity)

**Figure 1:**
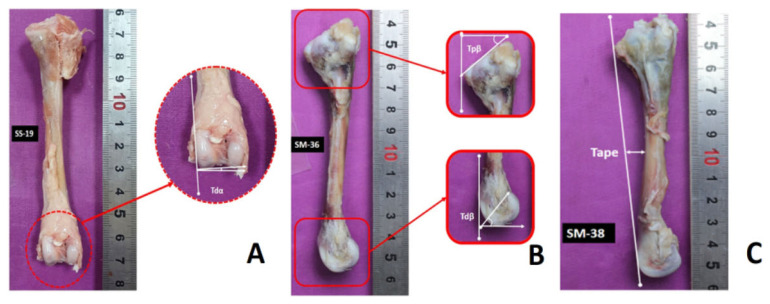
The analysis of varus-valgus deformity using solidworks software. (A) Showing tibial distal angulation, Tdα (right, cranial view); (B) showing tibial proximal bending, Tpβ; tibial distal bending, Tdβ; and (C) anterioposterior curvature, Tape (right, lateral view).

All the right tibias were photographed using a camera (EOS 550D; Canon, Tokyo, Japan). These images were taken in two directions (cranial and lateral surfaces). Later on, using solid works software (SOLIDWORKS, Waltham, MA, USA), the bones were analyzed for VV deformity. For this purpose, tibia distal angulation (Tdα), tibia proximal bending (Tpβ), tibia distal bending (Tdβ), and anteroposterior curvature (Tape) were measured (Karaarslan and Nazlıgül 2018) (Figure 1).

### Morphometric measurements

First of all, geometric measurements including bone weight (g) using a weighing balance (Densi/DS-10, ±1 g), bone length [12] proximal, middle (medial-lateral and cranio-caudal), distal diameters and right drumstick width with the help of digital calliper (Model: CD-15CP, CODE: 500-181-U; Mitutoyo, Kawasaki, Japan) with an accuracy of ±0.01 mm., were noted down for the right femora and tibiae [13].

Some indices were calculated as an indicator of whole bone density and strength. These were bone index (Seedor Index), robusticity index [14] and relative bone weights of the femora and tibiae using formulas [15] as given:


$$\begin{array}{l} \text{Bone index}=\text{Bone weight}/\text{Bone length}\\ \text{Robusticity index}=\text{Bone length}/\text{Cubic root of bone weight}\\ \text{Relative bone weight}=\text{Bone weight}/\text{Live weight}\times 100 \end{array}$$

### Biomechanical testing of the bone

After geometric calculations and photography, the right tibiae (n = 120) were subjected to a three-point bending test to assess biomechanical properties. A single-pronged loading device using Zwick/Roell mechanical test apparatus, Model: Z-0.5 T-Device, Germany (Zwick Roell, Ulm, Baden-Württemberg, Germnay) was applied at a mid-point between the two supports, and the span length was kept at 50 mm. The 2N pre-load and test speed of 10 mm/min were applied [16]. The testXpert II software was used to record the measured data. After the test application, data derived from the load-displacement curve (maximum force [F_max_] and displacement [d]) were used to calculate stiffness (S), the moment of inertia (I_x_), strength (δ), and elastic modulus (E), using various formulas [17].

### Ash analysis

After morphometry, the left femurs and tibias (n = 60) were subjected to an ashing process in a muffle furnace using the AOAC method. Firstly, bones were placed in chemicals (chloroform and ethanol) for 24 hours to remove fat. Then, fat-extracted weight was obtained after drying for one day at 60°C. Then, two partitions (division 1 and 2) of these femurs and tibias were made (10 bones each per group). Ten femurs and tibias, each per group, were ashed in a muffle furnace at 550°C for 6 h as a whole sample. The rest of the femurs and tibias (10 each per group) were cut up into epiphyseal and diaphyseal regions. The upper 25% and middle 10% of the length of the bone were designated as epiphysis and diaphysis of the bone, respectively. These bone sections were ashed in a muffle furnace at 600°C for 24 h [18] to get epiphyseal and diaphyseal ash separately for a sample. The data of the five best representative samples were given in the results section for ash percentage.


Ash%=[Total ash (g)×100]/Total weight of ground bone (g)

### Mineral composition

After ash determination for whole samples of bones (femurs and tibias), these samples were transferred to a digestion tube. Hydrochloric and nitric acid was added to it. Bone calcium (Ca), potassium (K), magnesium (Mg), and phosphorus (P) were analyzed using an ICP Mass Spectrometer (Model 3110, 1994; Perkin-Elmer, Waltham, MA, USA) at (The Agricultural Faculty of ADU) [19]. Five representative samples' data were provided afterwards.

### Statistical analysis

Statistical analysis was performed using the SPSS software package (version 22.0; SPSS Inc., Chicago, IL, USA). An analysis of variance (ANOVA) was conducted to compare the main effects and interaction of the type of litter used and live weight on different bone parameters. Both litters used and live weight included 3 levels of analysis: "ZL", "PF", and "SW". The data that were not normally distributed were log-transformed before the analysis. For the ordinal data of the TD score, frequencies and relative frequencies were obtained among groups, and multinomial logistic regression analysis was performed to determine the relationship between the TD and type of litter used and live weight. One-way ANOVA was used to analyze VV deformity, and the post-hoc Bonferroni test was applied to show the significance of litter on deformity by ANOVA. Spearman correlation tests were performed using R software between the live weight and type of litter and the parameters of bones to evaluate the potential contribution of live weight or litter. An overall correlation matrix of live weight and type of usage with different bone parameters and box and whisker diagram was created using statistical package R. The level of significance (α) was set at p<0.05.

## RESULTS

### Leg health indicators

Table 2 shows TD data and the distribution of scores 0 and 1 (normal and moderate). The chickens from PF and WS groups had a higher percentage of TD score = 1. Similarly, showed minor TD, and their leg health was poor. Zeolite bedding had the highest percentage of broiler with zero TD score (normal), though no bird with a TD score = 2 was observed in this study.

Multinomial logistic regression showed that bedding material (independent variable) had a significant (p = 0.038, R^2^ = 0.040) contribution towards the development of TD. The regression coefficient (B) for the litter group was 0.509. The exponential (B) value for TD in the litter group was 1.664 (95% CI: 1.028 to 2.695). Live weight could not imply any significant effect on the likelihood of TD in any group of the study (p = 0.527).

**Figure 2:**
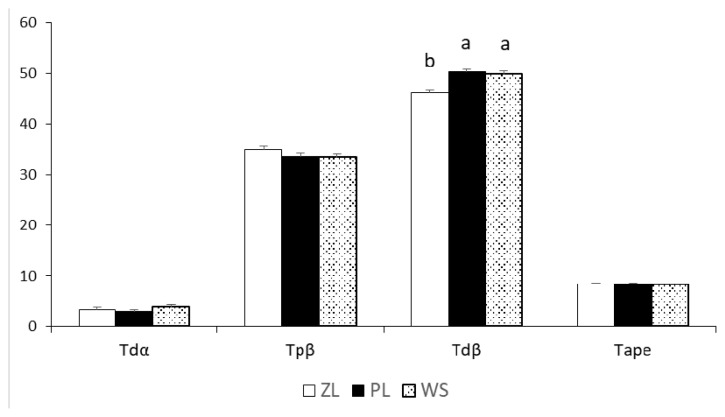
Represents the varus valgus (VV) deformities and the values for tibial distal angulations in different litter systems. Tdα, tibial distal angulation; Tpβ, tibial proximal bending; Tdβ, tibial distal bending; Tape, anterioposterior curvature. SEM, standard error of mean. ^a,b^ Means that do not share a common superscript differ significantly (p<0.05). ZL, zeolite-added litter; PF, plastic-grid flooring; WS, wood shavings.

The broiler bones were photographed for VV deformities and Tdα, Tpβ, Tdβ, and Tape are presented in Figure 2. Broilers raised on these three floorings were unaffected by VV deformity except the Tdβ, which was significantly higher in PF than the ZL group (p<0.001).

### Femur and tibial morphometric parameters

Femur bone weight was similar across the three groups, and femur length and robusticity index (RI) was lower in the PF group compared to the control flooring (p = 0.010, p = 0.048). RI in the WS group (31.99) was noted to be slightly higher than in the PF group (31.45, p = 0.048). The femur bone weight, length, and proximal and distal diameters were higher for the WS group (p = 0.030; 0.010; 0.001; <0.001). Chickens reared on zeolite litter had lower proximal and distal diameters of the femur bone (p = 0.001; <0.001). These significant differences in femur length, robusticity index, and proximal and distal diameters are shown in Table 3. Likewise, femur bone proximal and distal diameters were higher in the WS group, measured as 16.08 mm and 13.30 mm, respectively (p = 0.001; <0.001). Overall, the mean weights of tibial bones, proximal diameters and distal diameters are presented in Table 3, respectively. Zeolite flooring showed a significant effect on drumstick width (58.80 mm) compared with PF (53.56) and WS (56.81) groups (p = 0.001). However, no significant (p>0.05) difference was found for the tibial bone index, weight, robusticity index, relative weight, and proximal diameter (Table 3).

### Biomechanical properties of tibial bone

Various parameters, like displacement (d), M-L external diameter, stiffness (S), the moment of inertia (I_x_), and elastic modulus (E), were similar across the groups. Bone strength (δ) was highest in the ZL group (p = 0.040). The S and E values of the tibia did not differ significantly among groups, all the data related to bone biomechanics is presented in Table 4.

### Ash analysis

Total ash content, diaphyseal, and epiphyseal ash content of the femur differed among groups (Table 5). Femur total ash and epiphysis ash were higher in ZL compared with PF and WS groups (p = 0.044 and 0.016). Diaphysis ash and femur epiphysis contents were lowest in the PF group (p = 0.003 and 0.016). All other bone parameters for the femur and tibial mineral contents were not different among the groups.

### Correlation analysis of live weight and different bone properties in three groups

**Figure 3:**
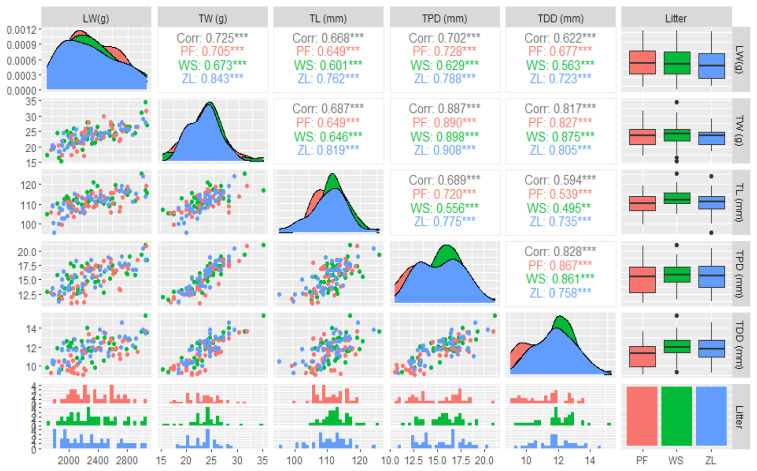
Scatter plots correlation matrix results for live weight and tibia bone parameters (weight, length, proximal and distal diameters) in three types of litter (PF, WS, ZL). ** p<0.05. Scatter plot correlation matrix of LW (live weight), TW (tibia weight), TL (tibia length), TP (tibia proximal diameter), and TD (tibia distal diameter) of broiler in three groups (PF, WS, ZL). The density plots on the diagonal axis show the density eclipses of different parameters. The lower off-diagonal section illustrates the histogram and the magnitude of the linear association between the variables. The right vertical section is displaying box and whisker plots. ZL, zeolite-added litter; PF, plastic-grid flooring; WS, wood shavings. The upper-diagonal section illustrates the significance of relationship between variables (** p<0.05).

Figure 3 shows Pearson correlation coefficients (r) with average live weight and tibia bone parameters for each type of litter (PF, WS, ZL). Tibia bone weight (g) had a strong correlation (r = 0.725, p<0.05) in all litter groups. ZL and PF had the strongest correlation (r = 0.843, 0.705, p<0.05), while WS was weaker (r = 0.673, p<0.05). Similarly, the tibia proximal diameter (mm) correlation across the litter groups (0.828, p<0.05) was stronger than the tibia distal diameter correlation, and each litter group analysis showed similar correlations (0.867, 0.861, 0.758), in plastic flooring, wood shaving and zeolite litter, respectively (p<0.05).

## DISCUSSION

The housing system plays a vital role in the leg health of broilers, as legs are in direct contact with the bedding material throughout the rearing period. In the present study, the overall live weight of broilers was less in the ZL group (p<0.05) which was reflected in the percentage of TD scores as well in respective weight categories (light, medium, heavyweight). Broiler chickens raised on PF, and WS litter had a higher percentage of medium and heavy-weight broilers, and a significant percentage of lightweight broilers was present in the ZL group. In addition, the incidence of TD was greater in PL and WS groups than in ZL (p<0.001). Our findings agree with Petek et al [20], in which various parameters like live weight, FCR, mortality, and dressing percentage were affected by the type of litter.

This study is the first of its kind to report biomechanical and morphometric changes together in the long bones of chickens. The tibia is widely considered an indicator of growth and skeletal health in poultry [21], and its shorter length shows a slower growth rate than in the ZL group. However, WS and PF groups had longer and heavier femur and tibia; thereby, the live weights were higher. Similar observations of longer tibia and humeri have been reported by Tolon and Yalcin [22] on floor pens compared with plastic mesh cages. The morphometric properties of long bones, i.e. femur and tibia, provide estimates of the skeletal development of broilers [23]. Skinner and Waldroup [24] recommended that the overall skeletal development of chickens should not be solely assessed by tibia only. Therefore, morphometric properties of the femur were also studied, as femur bone properties. Overall, the proximal and distal diameters of the femur and tibia of all birds followed a similar trend as the bone weight and length. However, femur proximal, distal diameters, and robusticity index were the lowest for the PF group. It has been documented that the husbandry system (cages, plastic mesh, floor pens) has a strong influence on bone morphometric properties [22] and skeletal morphology, and body weight [25]. These observations support the results of the present study, where bedding type significantly affected the geometric and biomechanical properties of both the femur and tibia. The Tibia bone has heavy muscle mass, directly supports body weight, and is most vulnerable to disorders in chickens [26]. Biomechanical tests revealed that the bone-breaking strength of the tibia was higher in birds of the ZL group. Although the numeric values of Fmax, d, and E were also higher for the ZL group, statistical difference was found only significant for the breaking strength of the tibia. Interestingly, the drumstick width and tibial breaking strength of ZL chickens were greater even though the birds were lightweight with shorter tibiae.

Furthermore, the tibia of ZL had similar proximal diameters as in WS and PF groups. This increase in bone width (diameter) compared to length might be the reason for greater bone-breaking strength in the ZL group. The supplementation of zeolites (100 g/kg) reduces litter moisture and ammonia levels, which might be another reason for bone strength and higher drumstick width [12]. It is also pertinent to mention that high locomotive activity and easy walking in ZL bedding could be attributable to high bone strength [27]. Total tibial bone ash contents were similar across the groups, and these findings agree with Tolon and Yalcin [22]. Vargas-Galicia et al [28] concluded that mineral contents (Ca, K, Mg, and P) of femur or tibia bones have no significant difference in different litter type groups. All these findings were further supported by ash analysis, and lower values of bone mineralization parameters (total ash, tibia and femur total, diaphyseal, and epiphyseal ash contents) were found in PF chickens. In fast-growing birds, cortical bone structures turn out to be porous [29], so lower elastic modulus and bone-breaking strength might be attributable to higher bone porosity in the PF group as it displayed the lowest mineralization.

The angles for evaluating VV deformity were within the normal range as reported in the literature. However, Tdβ values in PF and WS groups were higher than in ZL. In our observation, plastic-grid flooring allows a greater air passage, and heavyweight broilers prefer to lay on the floor for longer to alleviate heat stress. Therefore, heavyweight birds are the most affected ones, and their weak legs lose the ability to carry body weights beyond the normal limits; that's why Tdβ values were higher in PF broilers. Furthermore, leg deformities are related to poor walking ability, like a higher incidence of TD scores 1 and 2 in PF flooring (p<0.001) was found. Similar findings were reported by Kaukonen et al [30], where broiler birds were likely to develop severe TD on plastic floors in the absence of perches and bathing behaviour. Heitmann et al [7] documented that the moisture level in plastic flooring is similar to that of sawdust litter, so a higher incidence of dirty plumage and FPD is seen in PL floors. Plastic flooring reduces FPD scores in turkeys, but no improvement in broilers has been reported [31]. Similar results were obtained from Li et al [32], where netted floor increased the risk of breast blisters, and no differences in hock and foot pad lesions were found among different flooring systems.

Modern broilers spend approximately 80% of their time resting on litter, and this behaviour is a primary deterrent to hock burns and leg problems. Why some birds on the same floor type develop leg problems and others do not is further debatable, and it suggests a high individual variation among broiler chickens. In our study, the incidence of TD was higher in medium and heavy-weight birds of the PF group, as Kristensen et al [33] stated that heavyweight birds have an increased probability of lameness than lightweight birds. No work has been reported on the possible effect of zeolite litter on the incidence of TD and leg biomechanics. Eleroğlu and Yalçın [34], in their study, used zeolite (25% to 75%) in litter and found that it prevents bacterial proliferation, reduces foot abnormalities and moisture level (36.2% to 21.8%), and zeolite addition in the litter might be reasons for lower TD in our study. It has been documented that broilers choose bedding material close to a natural sandy environment [35] and prefer to dust bathe in sand litter than other types of bedding [36]. Our study observed that birds were more mobile and active in ZL and WS bedding environments, which were otherwise compromised in plastic-grid flooring. As wood shavings are close to the natural environment and a better option than rice husk bedding, reduced leg problems were seen in the WS group, as reported by Almeida Paz et al [36].

The bone strength is not determined only by its shape, mass, and length; architectural and material properties are also important to consider [37]. These good mechanical properties are acquired in a healthy environment with a good walking ability so that broilers can easily access the feeders and drinkers [38]. Perhaps, ZL birds experienced more mechanical adaptation compared to the birds of another flooring, and this could be considered as means of stronger bones. Similarly, elastic modulus (a material indicator of bone rigidity) was also higher in the ZL, showing that bone is less ductile and more rigid. The results of the mechanical study prove that broilers were comfortable on soft litter material (ZL) with sand-type quality rather than PF flooring with perforations.

Tibia bone indices like tibial weight, length, and proximal and distal diameter showed a strong positive association with the live weight of chickens. The weight of the tibia was strongly associated with proximal and distal diameters (r = 0.887, 0.828) and live weight (r = 0.817) of broilers in all three types of floorings. Interestingly, the live weight of chickens showed a positive correlation (r = 0.843) with the ZL group tibia bone weight (g). This clearly indicates that the tibia bone gives a direct idea of broiler growth and weight gain. Similarly, the highest positive association of tibia weight with the proximal diameter (r = 0.908) was also present in ZL. One more important finding is weak correlation of tibial bone length with distal diameter in WS and PF groups, but at the same time, ZL has a high positive value (r = 0.735). This observation agrees with the findings of Pedersen et al [39], where larger and longer bones were associated with better leg health in broilers.

## CONCLUSION

Leg deformities and bone problems are increasing in the poultry industry and cause major losses. Results of the study suggest that broilers raised on plastic flooring have higher weight gain, and the type of floor can contribute towards the incidence of TD. Tibia and femur bones are a good model for estimating bone and leg health in broilers, and this study also showed that tibial length and weight are strongly correlated with broiler live weight. Various bone strength parameters like tibial strength, drumstick width, femur and epiphyseal ash contents were comparatively better in broilers raised on zeolite litter than PF bedding. Although no litter is the best yet, maximum benefits can be obtained by replacing scarce and costly bedding. Zeolites can be a better bedding material for rearing broilers without compromising their skeletal properties and well-being. Further studies are necessary to validate whether flooring has a role in resting behaviour, the development of leg disorders, and the relationship between tibia bone strength and the time broilers spend resting and lying.

## Figures and Tables

**Table 1:** Experimental design

Trial groups	Bedding material	Sub groups	Birds per pen	Total per group
Group I (ZL)	Zeolite	4	42	168
Group II (PF)	Plastic-grid	4	42	168
Group III (WS)	Wood shavings	4	42	168
Total birds				504

ZL, zeolite-added litter; PF, plastic-grid flooring; WS, wood shavings.

**Table 2:** Descriptive statistics showing frequencies of TD in broilers (n = 120)

Live weight	TD score	Groups			X^2^	p-value

		ZL	PF	WS		
Light-weight	0	10 (83.4%)^a^	1 (8.3%)^b^	1 (8.3%)^ab^	7.708	0.021
	1	0 (0%)^a^	2 (66.7%)^b^	1 (33.3%)^ab^		
	2	0 (0%)^a^	0 (0%)^a^	0 (0%)^a^		
Medium-weight	0	18 (56.3%)^a^	10 (31,3%)^b^	4 (12.4%)^b^	19.095	<0.001
	1	3 (9.7%)^a^	21 (67.7%)^b^	7 (22.6%)^b^		
	2	0 (0%)^a^	3 (60.0%)^a^	2 (40.0%)^a^		
Heavy-weight	0	5 (27.8%)	8 (44.4%)	5 (27.8%)	2.107	0.716
	1	4 (25.0%)	7 (43.8%)	5 (31.2%)		
	2	0 (0%)	2 (66.7%)	1 (33.3%)		

TD, tibial dyschondroplasia; ZL, zeolite-added litter; PF, plastic-grid flooring; WS, wood shavings.

a,b Means within a row that do not share a common superscript differ significantly (p<0.05).

**Table 3:** Effect of type of litter on femur and tibia traits of 42-day old broiler chickens (means)

Parameters		ZL	PF	WS	p-value
Live weight (g)		2,239.55±51.95^b^	2,406.53±47.56^a^	2,438.88±51.86^a^	0.013
Weight (g)	Femur	16.86± 0.30^b^	17.33±0.33^ab^	17.88±0.40^a^	0.030
	Tibia	23.28±0.49	23.38±0.63	23.89±0.56	0.658
Length (mm)	Femur	81.74±0.54^b^	81.02±0.50^b^	83.25±0.49^a^	0.010
	Tibia	110.69±0.94	110.81±0.75	112.93±0.66	0.070
Bone index	Femur	0.20±0.00	0.21±0.00	0.21±0.00	0.263
	Tibia	0.21±0.00	0.21±0.00	0.21±0.00	0.971
Relative weight	Femur	0.74±0.01	0.73±0.01	0.76±0.02	0.158
	Tibia	1.02±0.01	0.995±0.02	1.02±0.02	0.428
Robusticity index	Femur	31.82±0.13^a^	31.45±0.15^b^	31.99±0.18^a^	0.048
	Tibia	38.84±0.18	38.90±0.25	39.38±0.25	0.163
Proximal diameter (mm)	Femur	14.59±0.28^b^	14.51±0.30^b^	16.08±0.39^a^	<0.001
	Tibia	15.53±0.40	15.08±0.47	15.76±0.38	0.415
Distal diameter (mm)	Femur	11.76±0.19^b^	11.38±0.21^b^	13.30±0.32^a^	<0.001
	Tibia	11.81±0.22^a^	11.21±0.26^b^	11.93±0.22^a^	0.030
Right drumstick width (mm)	Tibia	58.80±0.76^a^	53.56±1.22^b^	56.81±0.87^a^	0.001

ZL, zeolite-added litter; PF, plastic-grid flooring; WS, wood shavings.

a,b Means within a row that do not share a common superscript differ significantly (p<0.05).

**Table 4:** Showing biomechanical properties of tibiotarsal bone

Biomechanical parameters	ZL	PF	WS	SEM	p-value
F_max_ (N)	301.98	281.47	294.65	5.50	0.248
d	4.58	4.47	4.45	0.05	0.492
M-L external diameter (mm)	9.22	9.22	9.35	0.07	0.699
I_X_ (m^4^)	1.99E-10	1.98E-10	2.26E-10	8.48E-12	0.289
δ (MPa)	84.01^a^	74.87^b^	74.19^b^	1.72	0.040
S (N/mm)	91.08	90.66	94.31	1.49	0.502
E (MPa)	1,435.82	1,314.03	1,326.82	55.78	0.647

ZL, zeolite-added litter; PF, plastic-grid flooring; WS, wood shavings; SEM, standard error of means; F_max_, maximum force; d, displacement; I_x_, moment of inertia; δ, strength; S, stiffness; E, elastic modulus.

a,b Means within a row that do not share a common superscript differ significantly (p<0.05).

**Table 5:** Showing percentage of ash and mineral composition of femur and tibia (broiler chickens) on different types of litter

Items		ZL	PF	WS	SEM	p-value
Total ash (%)	Femur	53.64^a^	50.91^b^	52.90^b^	0.40	0.044
	Tibia	54.32	53.82	55.58	0.33	0.122
Diaphysis aAsh (%)	Femur	63.31^b^	60.86^c^	63.69^a^	0.28	0.003
	Tibia	64.86	64.05	64.31	0.21	0.318
Epiphysis ash (%)	Femur	50.90^a^	47.23^c^	48.72^b^	0.44	0.016
	Tibia	47.30	49.07	49.50	0.66	0.280
Ca (%)	Femur	16.50	15.67	18.21	0.56	0.207
	Tibia	16.02	16.07	16.57	0.52	0.894
K (%)	Femur	5.73	5.44	5.67	0.36	0.940
	Tibia	5.33	4.11	5.58	0.33	0.191
Mg (%)	Femur	5.59	5.58	5.80	0.12	0.726
	Tibia	5.58	5.37	5.51	0.09	0.662
P (%)	Femur	7.42	7.37	7.60	0.17	0.859
	Tibia	7.77	7.75	7.43	0.22	0.775

Femur and tibia ash% and minerals in the columns and rows show different types of litter (ZL, PF, and WS groups).

ZL, zeolite-added litter; PF, plastic-grid flooring; WS, wood shavings; SEM, standard error of means.

a–c Means within a row that do not share a common superscript differ significantly (p<0.05).

## References

[1] Bordoni A, Danesi F, Petracci M, Berri C (2017). Chapter 11 - Poultry meat nutritive value and human health. Woodhead Publishing Series in Food Science, Technology and Nutrition: poultry quality evaluation.

[2] Atapattu NSBM, Wickramasinghe K (2007). The use of refused tea as litter material for broiler chickens. Poult Sci.

[3] Mumpton FA, Fishman PH (1977). The application of natural zeolites in animal science and aquaculture. J Anim Sci.

[4] Garcia R, Almeida Paz I, Caldara FR (2012). Litter materials and the incidence of carcass lesions in broilers chickens. Bras J Poult Sci.

[5] Granquist EG, Vasdal G, De Jong IC, Moe RO (2019). Lameness and its relationship with health and production measures in broiler chickens. Animal.

[6] Petek M, Sönmez G, Yildiz H, Baspinar H (2005). Effects of different management factors on broiler performance and incidence of tibial dyschondroplasia. Br Poult Sci.

[7] Heitmann S, Stracke J, Adler C (2020). Effects of a slatted floor on bacteria and physical parameters in litter in broiler houses. Vet Anim Sci.

[8] Van der Pol CW, Molenaar R, Buitink CJ (2015). Lighting schedule and dimming period in early life: consequences for broiler chicken leg bone development. Poult Sci.

[9] Buijs S, Van Poucke E, Van Dongen S, Lens L, Baert J, Tuyttens FA (2012). The influence of stocking density on broiler chicken bone quality and fluctuating asymmetry. Poult Sci.

[10] Erdal R, Richardson I, Ljøkjel K, Haug A (2012). Sensorial quality and bone strength of female and male broiler chickens are influenced by weight and growth rate. Br poult Sci.

[11] Wabeck CJ, Littlefield LH (1972). Bone strength of broilers reared in floor pens and in cages having different bottoms. Poult Sci.

[12] Schneider AF, Almeida DS, Yuri FM, Zimmermann OF, Gerber MW, Gewehr CE (2016). Natural zeolites in diet or litter of broilers. Br Poult Sci.

[13] Kolakshyapati M, Flavel RJ, Sibanda TZ, Schneider D, Welch MC, Ruhnke I (2019). Various bone parameters are positively correlated with hen body weight while range access has no beneficial effect on tibia health of free-range layers. Poult Sci.

[14] Azad S, Shariatmadari F, Torshizi M, Chiba L (2020). Comparative effect of zinc concentration and sources on growth performance, accumulation in tissues, tibia status, mineral excretion and ımmunity of broiler chickens. Braz J Poult Sci.

[15] Salaam ZK, Akinyemi MO, Osamede OH (2016). Biotechnology in Animal Husbandry. Effect of strain and age on bone integrity of commercial broiler chickens. Biotechnol Anim Husbandary.

[16] Komal K, Kilimci FS, Mehmet K (2021). Biomechanical tests: applications and their reliability for the prediction of bone strength in broiler chicken. Vet J Mehmet Akif Ersoy Univ.

[17] Jepsen KJ, Silva MJ, Vashishth D, Guo XE, Van Der Meulen MCH (2015). Establishing biomechanical mechanisms in mouse models: practical guidelines for systematically evaluating phenotypic changes in the diaphyses of long bones. J Bone Miner Res.

[18] Choi WJ, Kim JH, Han GP, Kwon CH, Kil DY (2021). Effects of dietary hatchery by-products on growth performance, relative organ weight, plasma measurements, immune organ index, meat quality, and tibia characteristics of broiler chickens. Anim Biosci.

[19] Moyo S, Jaja IF, Mopipi K, Masika P, Muchenje V (2021). Effect of graded levels of Imbrasia belina meal on blood lipid profile, bone morphometric and mineral content of broiler chickens. Anim Feed Sci Technol.

[20] Petek M, Üstüner H, Yeşilbağ D (2014). Effects of stocking density and litter type on litter quality and growth performance of broiler chicken. Kafkas Univ Vet Fak Derg.

[21] Jendral MJ, Korver DR, Church JS, Feddes JJR (2008). Bone mineral density and breaking strength of white leghorns housed in conventional, modified, and commercially available colony battery cages. Poult Sci.

[22] Tolon B, Yalcin S (1997). Bone characteristics and body weight of broilers in different husbandry systems. Br Poult Sci.

[23] Moran ET, Todd MC (1994). Continuous submarginal phosphorus with broilers and the effect of preslaughter transportation: Carcass defects, further-processing yields, and tibia-femur integrity. Poult Sci.

[24] Skinner J, Waldroup P (1995). Allometric bone development in floor-reared broilers. J Appl Poult Res.

[25] Yair R, Uni Z, Shahar R (2012). Bone characteristics of late-term embryonic and hatchling broilers: Bone development under extreme growth rate. Poult Sci.

[26] Harash G, Richardson KC, Alshamy Z (2020). Basic morphometry, microcomputed tomography and mechanical evaluation of the tibiotarsal bone of a dual-purpose and a broiler chicken line. PLoS One.

[27] Türkyilmaz MK, Nazligül A, Fİdan ED, Karaarslan S, Kaya M, Kİlİmcİ FS (2020). The effect of perch cooling and perch height on some bone strength parameters in broilers reared in summer. Harran Üniv Vet Fak Derg.

[28] Vargas-Galicia AJ, Sosa-Montes E, Rodríguez-Ortega LT (2017). Effect of litter material and stocking density on bone and tendon strength, and productive performance in broilers. Can J Anim Sci.

[29] Mignon-Grasteau S, Chantry-Darmon C, Boscher MY (2016). Genetic determinism of bone and mineral metabolism in meat-type chickens: a QTL mapping study. Bone Rep.

[30] Kaukonen E, Norring M, Valros A (2017). Perches and elevated platforms in commercial broiler farms: use and effect on walking ability, incidence of tibial dyschondroplasia and bone mineral content. Animal.

[31] Chuppava B, Visscher C, Kamphues J (2018). Effect of different flooring designs on the performance and foot pad health in broilers and turkeys. Animals.

[32] Li H, Wen X, Alphin R, Zhu Z, Zhou Z (2017). Effects of two different broiler flooring systems on production performances, welfare, and environment under commercial production conditions. Poult Sci.

[33] Kristensen HH, Perry GC, Prescott NB, Ladewig J, Ersbøll AK, Wathes CM (2006). Leg health and performance of broiler chickens reared in different light environments. Br Poult Sci.

[34] Eleroğlu H, Yalçın H (2005). Use of natural zeolite-supplemented litter increased broiler production. S Afr J Anim Sci.

[35] Shields SJ, Garner JP, Mench JA (2005). Effect of sand and wood-shavings bedding on the behavior of broiler chickens. Poult Sci.

[36] Almeida Paz ICL, Garcia RG, Bernardi R (2010). Selecting appropriate bedding to reduce locomotion problems in broilers. Braz J Poult Sci.

[37] Rath NC, Huff GR, Huff WE, Balog JM (2000). Factors regulating bone maturity and strength in poultry. Poult Sci.

[38] Kestin SC, Su G, Sorensen P (1999). Different commercial broiler crosses have different susceptibilities to leg weakness. Poult Sci.

[39] Pedersen IJ, Tahamtani FM, Forkman B, Young JF, Poulsen HD, Riber AB (2020). Effects of environmental enrichment on health and bone characteristics of fast growing broiler chickens. Poult Sci.

